# Supplementary figures and images for: Use of extracellular vesicle microRNA profiles in patients with acute myeloid leukemia for the identification of novel biomarkers
Source: PLoS One. 2024 Aug 23;19(8):e0306962. doi: 10.1371/journal.pone.0306962 (PMC11343415; doi:10.1371/journal.pone.0306962)

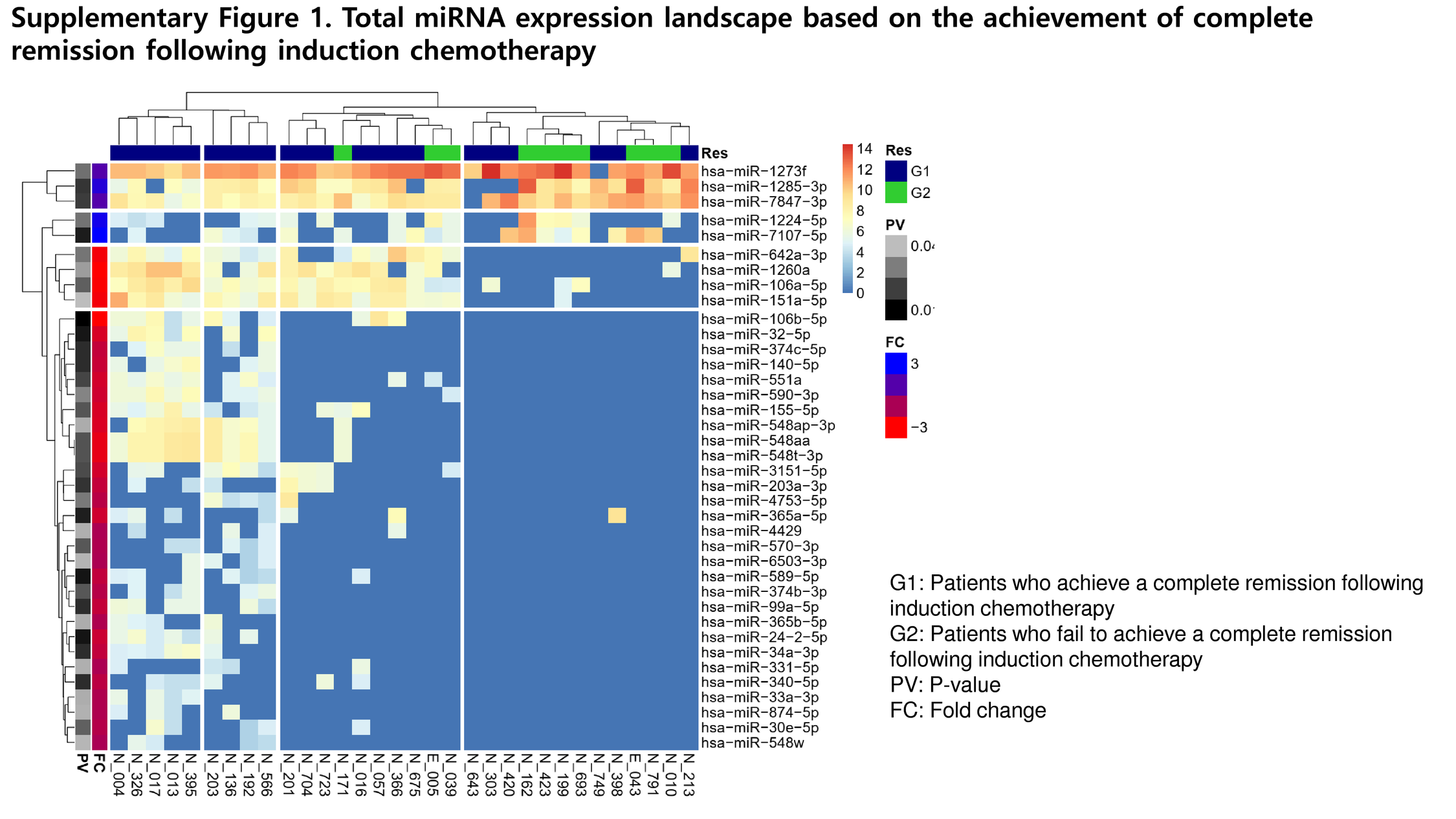

Supplement: S1 Fig — G1: Patients who achieve a complete remission following induction chemotherapy. G2: Patients who fail to achieve a complete remission following induction chemotherapy. PV, P-value; FC, Fold change. (TIF) [file pone.0306962.s001.tif]

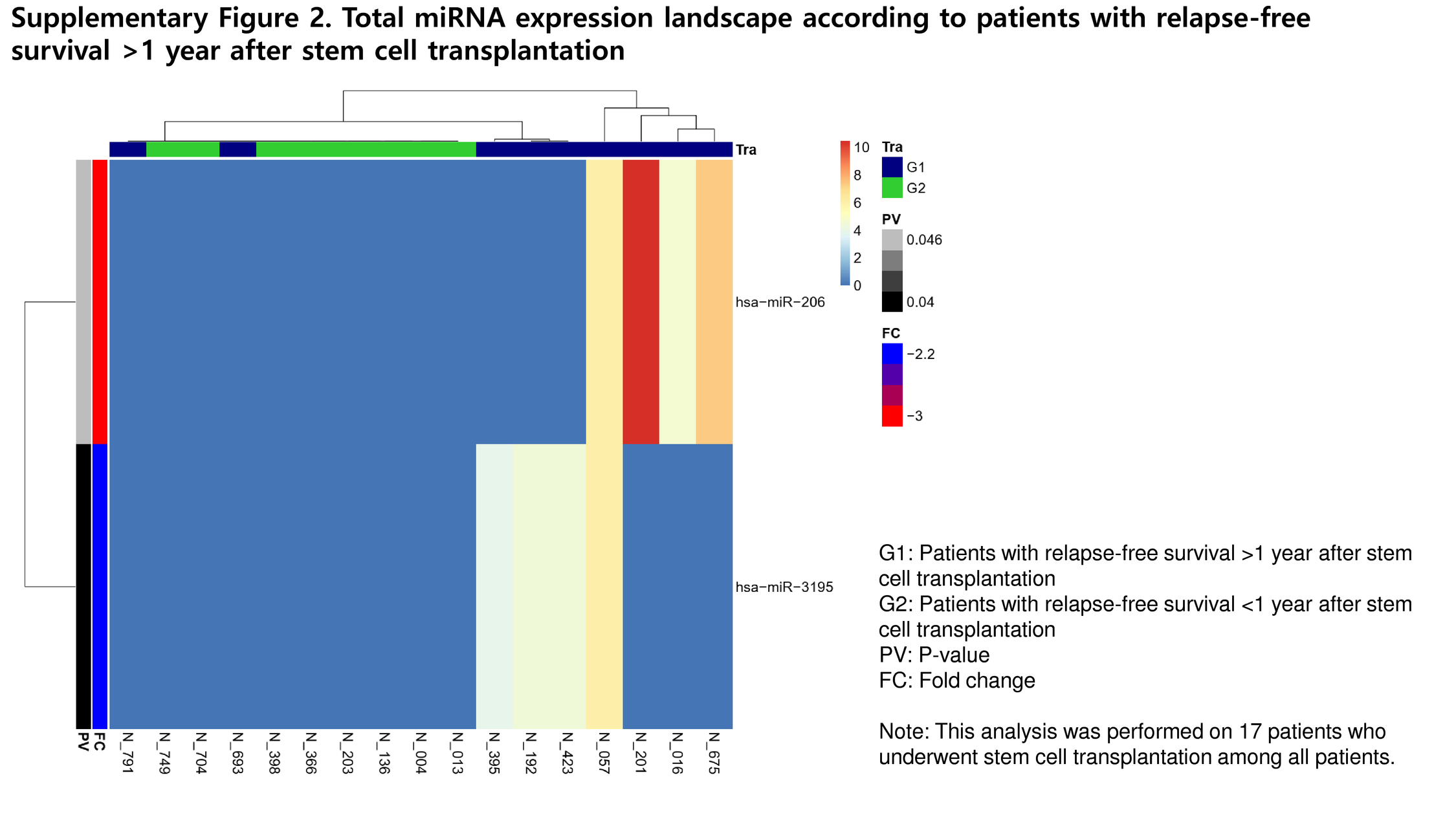

Supplement: S2 Fig — G1: Patients with relapse-free survival >1 year after stem cell transplantation. G2: Patients with relapse-free survival <1 year after stem cell transplantation. PV, P-value; FC, Fold change. Note: This analysis was performed on 17 patients who underwent stem cell transplantation among all patients. (TIF) [file pone.0306962.s002.tif]

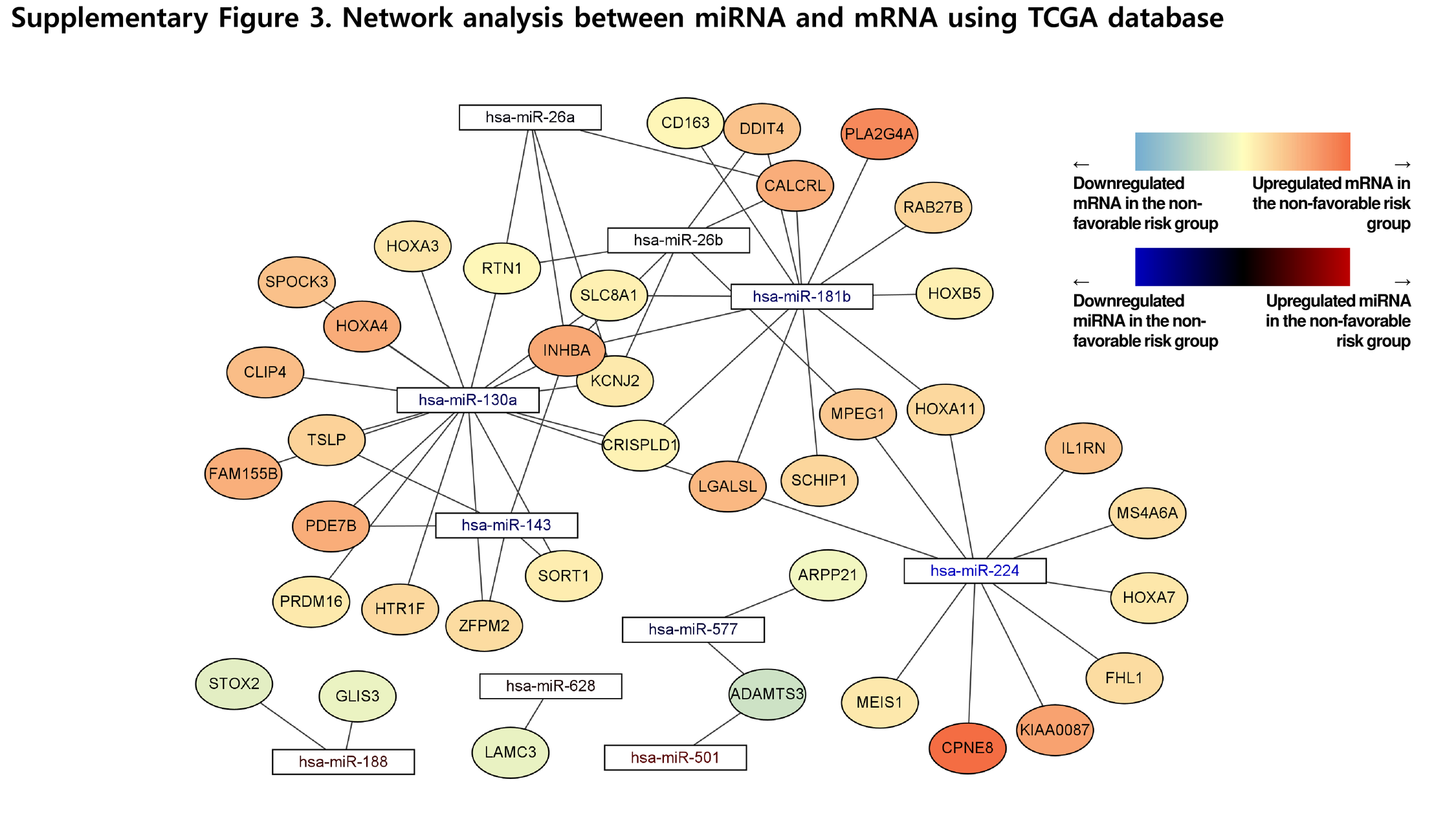

Supplement: S3 Fig — miRNA, microRNA; TCGA, The Cancer Genome Atlas. (TIF) [file pone.0306962.s003.tif]

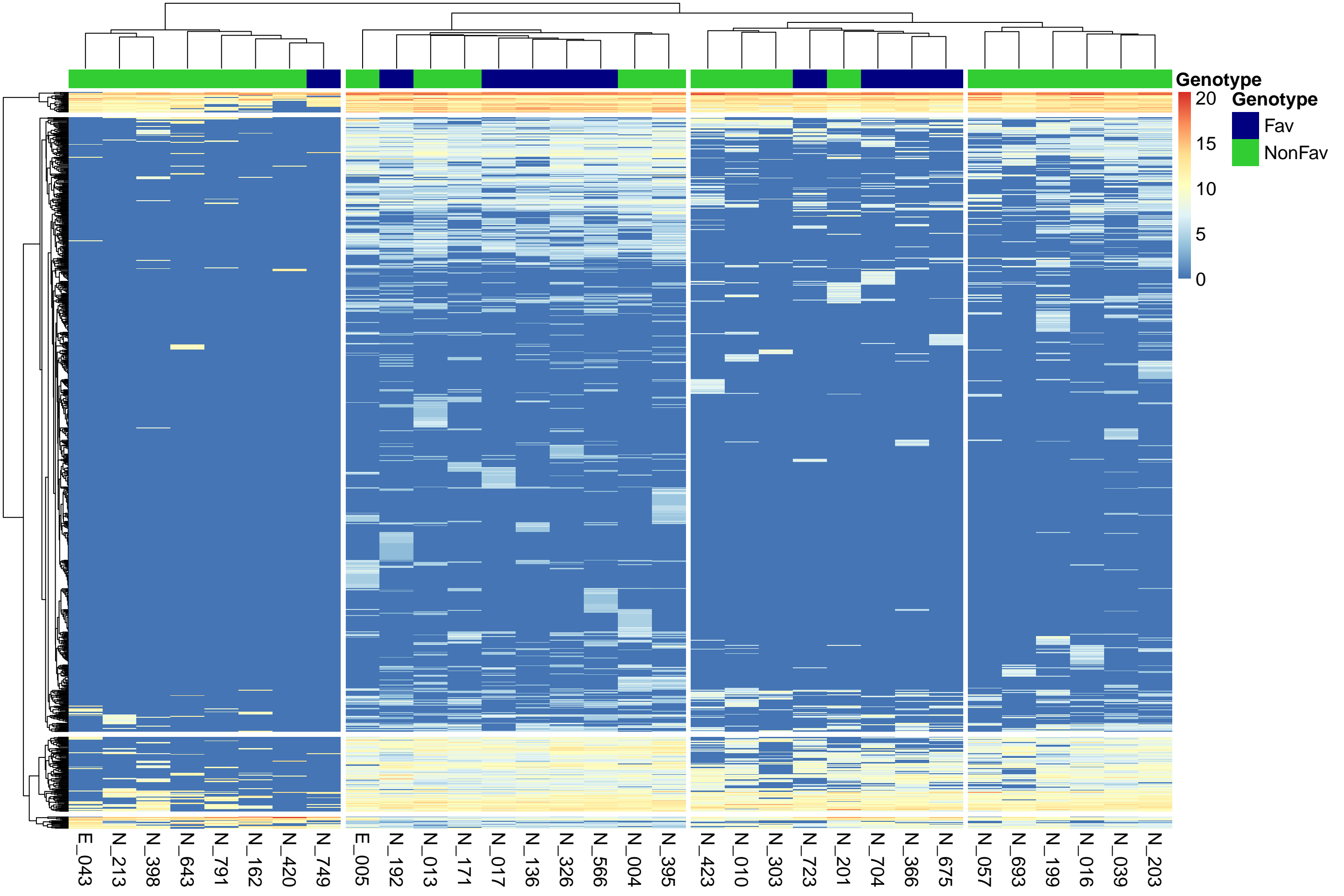

Supplement: S2 File — (PDF) [file pone.0306962.s006.pdf]
