# Supplementary material for: Use of extracellular vesicle microRNA profiles in patients with acute myeloid leukemia for the identification of novel biomarkers
Source: PLoS One. 2024 Aug 23;19(8):e0306962. doi: 10.1371/journal.pone.0306962 (PMC11343415; doi:10.1371/journal.pone.0306962)

** All images were acquired using an Amersham ImageQuant 800 Western blot imaging system (Cytiva, Little Chalfont, UK).

170 sec (CD63), 1st Serum, 2nd Plasma

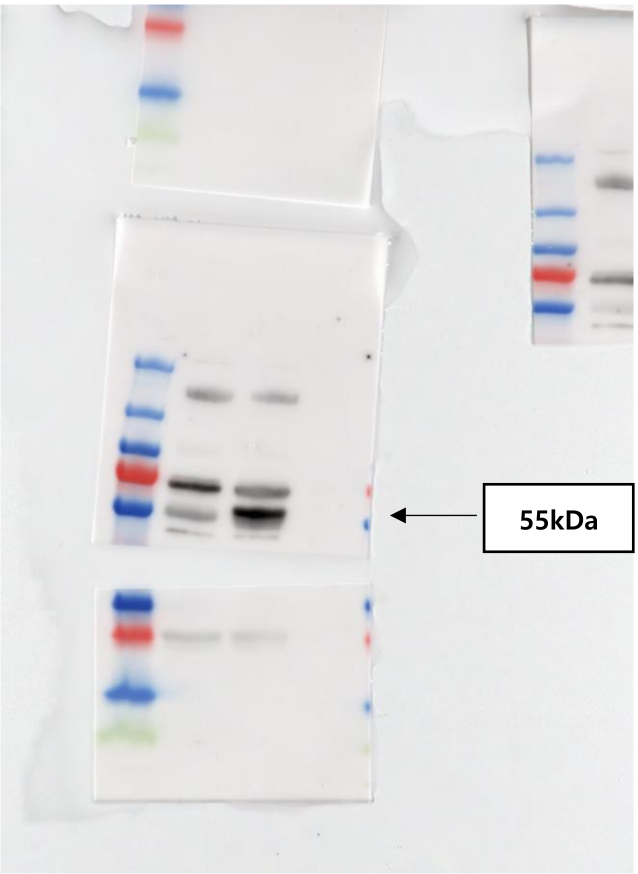

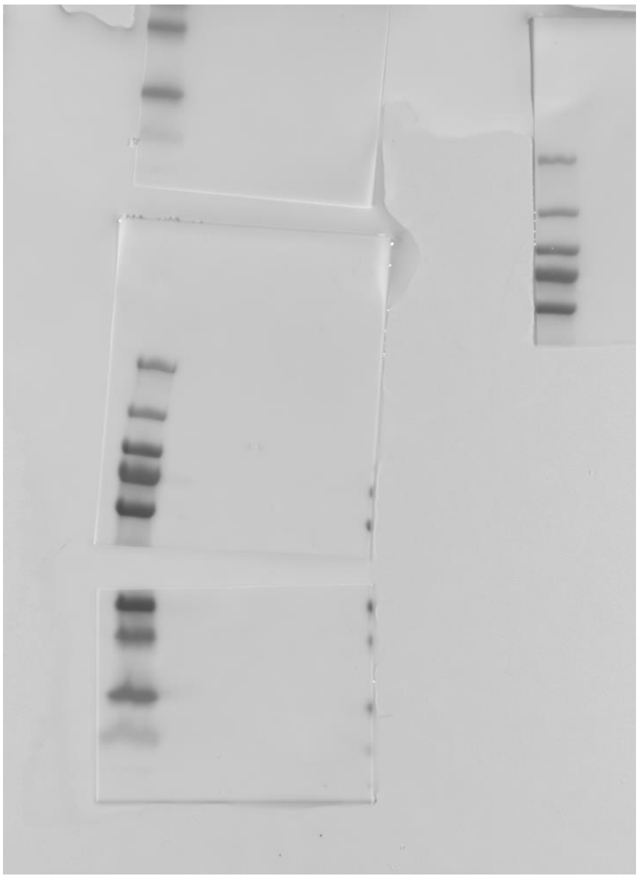

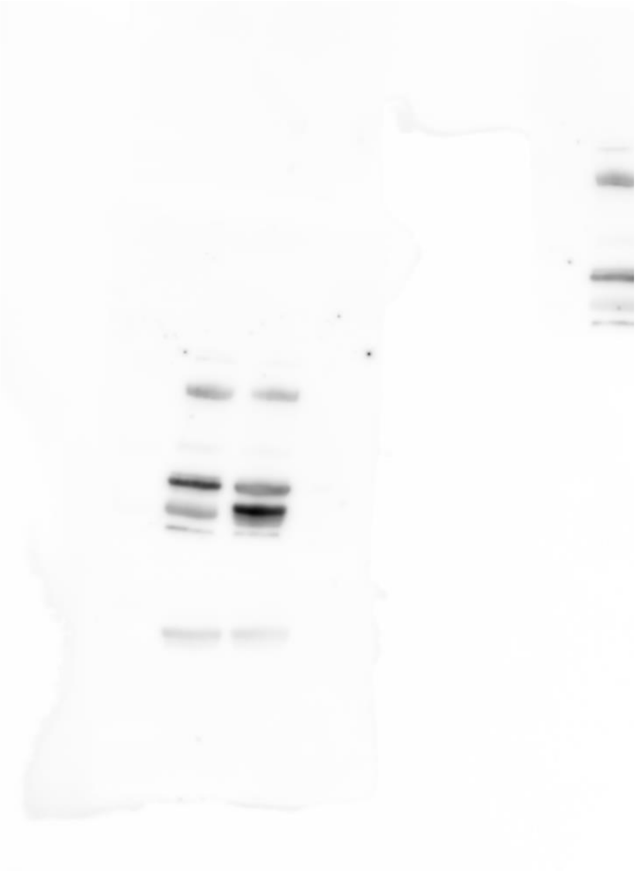

12min (CD81), 1st Serum, 2nd Plasma

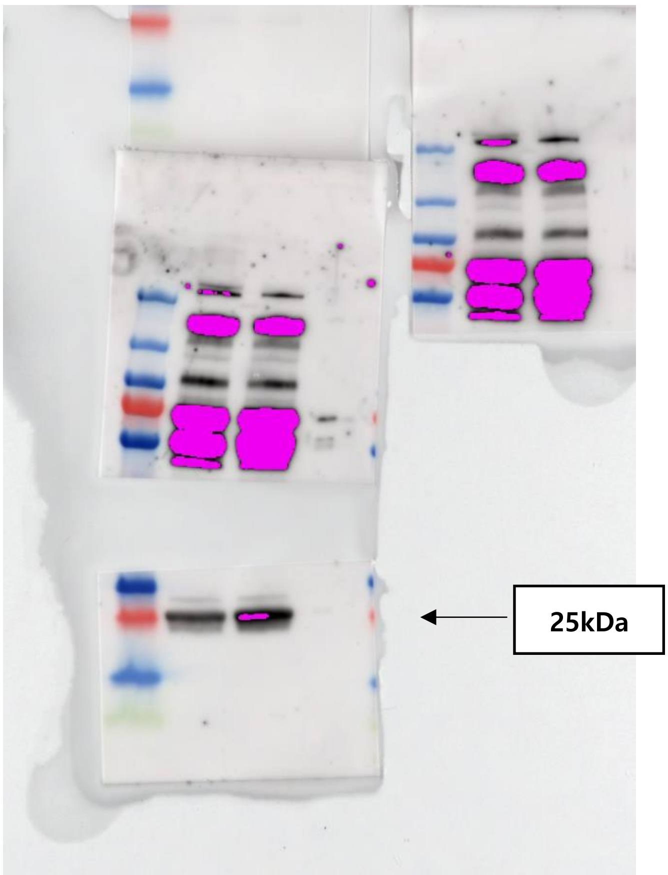

25kDa

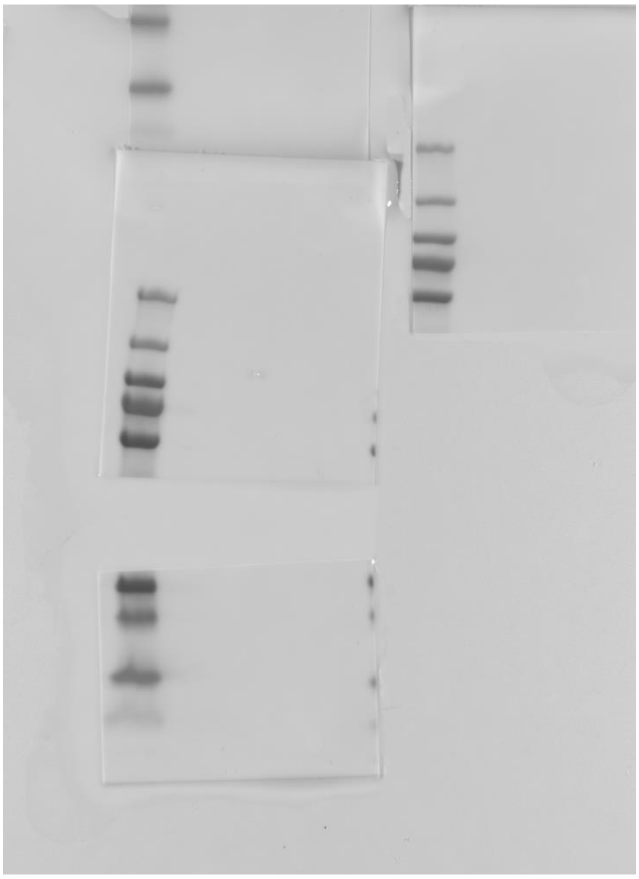

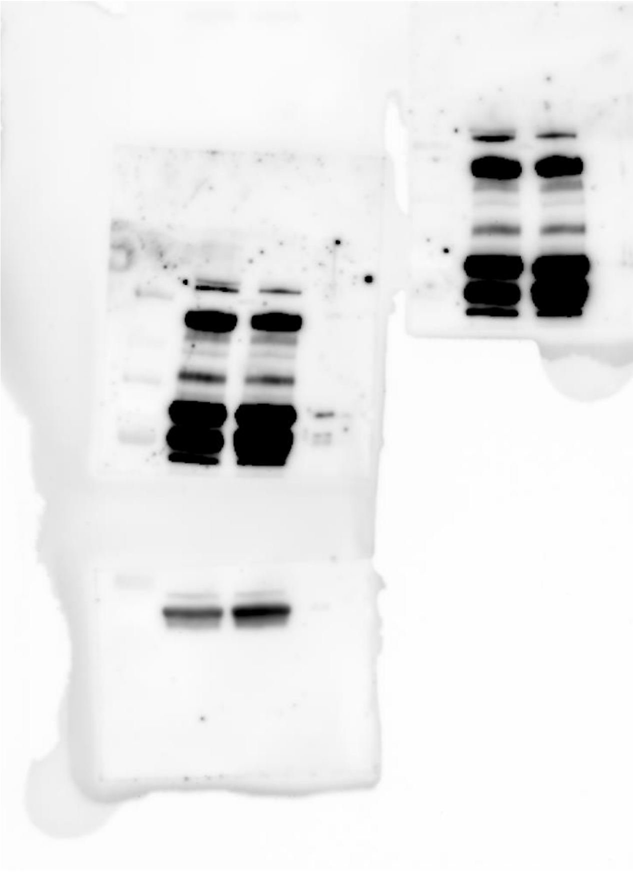

Supplement: S1 Raw images — (PDF) [file pone.0306962.s004.pdf]
