# Supplementary material for: Use of extracellular vesicle microRNA profiles in patients with acute myeloid leukemia for the identification of novel biomarkers
Source: PLoS One. 2024 Aug 23;19(8):e0306962. doi: 10.1371/journal.pone.0306962 (PMC11343415; doi:10.1371/journal.pone.0306962)
Supplement: Attachment — Submitted filename: 0_Response_to_reviewers_20240328.docx [file pone.0306962.s010.docx]

**Manuscript ID: PONE-D-23-13124**

**Title:** Use of extracellular vesicle microRNA profiles in patients with acute myeloid leukemia for the identification of novel biomarkers

Dear Prof. George Vousden:

Thank you for your help in processing our manuscript, and we appreciate the reviewers’ thoughtful comments. First, we have formatted the manuscript according to the style requirements of PLOS ONE and modified content regarding the Data Availability statement and ethics statement. Unadjusted blot images were uploaded in Supporting Information files. We have also revised the manuscript in accordance with the reviewers’ comments and suggestions. Point-by-point responses are included in this letter to each comment of the reviewers. Our revisions address all points raised by the reviewers. Additionally, our manuscript has been reviewed by an English-speaking editor.

We believe that changes made in response to the reviewers’ comments have significantly improved the revised manuscript, and we hope that the revised manuscript is now suitable for publication.

Sincerely yours,

Yong Park, MD, Ph.D.

Department of Internal Medicine, Korea University College of Medicine 73 Goryeodae-ro, Seongbuk-gu, Seoul 02841, Republic of Korea

Tel: +82-2-920-6847, Fax: +82-2-920-6520, E-mail: [paark76@hanmail.net](mailto:paark76@hanmail.net)

**List of reviewers’ comments and point-by-point responses**

**In each response, page and line numbers refer to the revised manuscript.**

**In the revised manuscript, text changes are highlighted in yellow.**

**Reviewer #1:**

The Authors presented interesting results about differentially expressed vesicular miRNA that may serve as risk markers in adult AML patients. The Authors focused on EVs circulating in the BM and performed the analysis by NGS. I have some comments:

**1. AML are conventionally considered a group of disorders, so I suggest the Authors to refer to AML as plural.**

***Response***: We appreciate the reviewer's valuable feedback. While we acknowledge that acute myeloid leukemia (AML) is conventionally considered a group of disorders, it is common practice in literature to refer to AML in the singular form. This is in line with established usage and helps maintain clarity in communication. However, we have provided appropriate context and clarification when discussing multiple instances or cases of AML in the manuscript.

**2. How were the samples stored? Was the BM stored post centrifugation (meaning BM plasma) or as whole blood?**

***Response***: Thank you for your careful review of our manuscript. We have incorporated your recommendations in the manuscript (Page 7, lines 3–12).

**3. Page 12. The Authors reported 2 average sizes of BM derived vesicles, but only 1 cohort is present. What do they mean? What does "respectively" refer to?**

***Response***: Thank you very much for your thoughtful comment. We used commercial size-exclusion chromatography to isolate EVs. While this method is applicable to both patient serum and plasma, there may be variations. Therefore, we presented separated EV profiles when using serum and plasma from a representative patient sample using size-exclusion chromatography. Additional clarification was included in the manuscript (Page 14, lines 4–11).

**4. Why did not the Authors consider also the miRNA differentially expressed in the favourable group as positive markers?**

***Response***: Thank you for your valuable comment. Risk stratification in AML plays a critical role in determining treatment strategies and predicting outcomes. It is well known that favorable-risk AML has low relapse and high survival rates with induction and consolidation chemotherapy alone, without the need for stem cell transplantation. On the other hand, in intermediate- or adverse-risk AML, the consideration of stem cell transplantation is essential in the treatment decision. Therefore, in this study, we categorized the groups as favorable and non-favorable, according to the different treatment approaches in clinical practice. We added this content to the manuscript (Page 7, lines 13–23).

**5. I strongly suggest to analyze the correlation between the differentially expressed miRNA and the type of chemotherapy. Moreover, I am curious to investigate when the success of allogeneic transplant correlates with some altered expression of vesicular miRNA.**

***Response***: We thank you for your valuable comments. The samples analyzed in this study were collected from bone marrow at the time of diagnosis of AML prior to induction chemotherapy. Therefore, these samples may not be suitable for analyzing the differential expression of miRNAs based on the type of chemotherapy used or the transplantation success. We will consider your suggestions for future research efforts.

**6. In the discussion section, please add some comments concerning the application of the presented findings in translational medicine for liquid biopsy purpose. Some other Groups suggested to search leukemic markers in EVs of patients with AML, such as PMID 36263223, PMID 37536029, PMID 36926576, and PMID 36139669. Please, add a comment on these papers.**

***Response***: Thank you for your suggestion.

- In accordance with the recommendations provided, we added relevant content to the Discussion section (Page 28, lines 17–23, and Page 29, lines 1–14). The role of EVs in the crosstalk between AML and the microenvironment and the possibilities of liquid biopsy were mentioned with references to the recommended studies (PMID 37536029, PMID 36926576, and PMID 36139669).

- In the limitations section citing your recommended study (PMID 36263223), we mentioned the limitations of interpreting results based on the EV separation method (Page 30, lines 13–20).

**7. Finally I suggest the Authors to stress why are they searching miRNA within the EVs released by cells that are present in the same samples (I mean, BM samples).**

***Response***: Thank you for your suggestion. In accordance with the recommendations provided, we added relevant content to the Discussion section (Page 29, lines 7–14).

**Reviewer #2:**

In this study, the authors establish clinically significant microRNA sets using EVs from BM aspirates of patients with AML, and to identify the genes that interact with these EVs derived miRNAs in AML. They findings of this study revealed that EV-derived miRNAs (hsa-mir-181b, hsamir-143, hsa-mir-130a, hsa-mir-224, hsa-mir-188, and hsa-mir-501) may be biomarkers for risk stratification and prognosis in AML. In summary, there are several issues that require attention before the manuscript is considered ready for publication. The following is a list, not prioritized by importance, of these concerns.

**1. Introduction section**

**(1) …accumulation of somatically acquired genetic alterations… include a reference**

***Response***: Thank you for your careful review of our manuscript. In accordance with the recommendations provided, references were added (Page 5, line 16).

**(2) …and may be useful biomarkers for the early detection of AML recurrence or prognosis… include a reference**

***Response***: Thank you for your careful review of our manuscript. In accordance with the recommendations provided, references were added (Page 6, line 5).

**(3) ….used EV isolation methods, such as sequential ultracentrifugation or precipitation**

**methods, with a relatively low purity….include a reference**

***Response***: Thank you for your careful review of our manuscript. In accordance with the recommendations provided, references were added (Page 6, line 16).

**2. Review the abbreviations throughout the document: EV or EVs**

***Response***: Thank you for your careful review of our manuscript. As per your recommendation, the entire manuscript has been re-evaluated for abbreviations.

**3. The authors could provide more information regarding the methods employed to determine the risk stratification and overall survival of the samples.**

***Response***: Thank you for your careful review. We provided a detailed explanation of the criteria used for risk stratification and the rationale behind categorizing it into favorable and non-favorable (intermediate and adverse) groups (Page 7, lines 13–23). Furthermore, we incorporated the overall survival data for the samples in **Table 1**.

**4. Specify the method for quantifying the bands in the Western Blot**

***Response***: Thank you for your careful review of our manuscript. The information was added to the manuscript (Page 10, lines 13–14, and page 10, line 20–23).

**5. Results Section, Table 1: Abbreviations such as EVs, BM... are not found within the table**

***Response***: Thank you for your careful review of our manuscript. We reviewed the relevant content and removed unnecessary abbreviations as per your instructions.

**6. Discussion section**

**(1) …between downregulated miR-181b expression and poor prognosis in patients with AML…… include a reference.**

***Response***: Thank you for your careful review of our manuscript. In accordance with the recommendations provided, references were added (Page 28, line 2).

**(2) …The central role of miRNAs is to regulate gene expression through canonical and non-canonical mechanisms…… include a reference**

***Response***: Thank you for your careful review of our manuscript. In accordance with the recommendations provided, references were added (Page 30, line 22).

**(3) The sentence: “This study aimed to report not only the significance of EV-derived miRNA as a biomarker, but also to identify mRNAs with potential as biomarkers”…should go at the beginning of the discussion section**

***Response***: Thank you for your valuable comment. In accordance with the recommendation provided, we relocated the corresponding sentence to the beginning of the Discussion section (Page 27, lines 8–10).

**(4) …Several studies have reported a correlation between downregulated miR-181b expression and poor prognosis in patients with AML… include a reference.**

***Response***: Thank you for your careful review of our manuscript. In accordance with the recommendations provided, references were added (Page 28, line 2).

**7. The quality of presented EVs images needs enhancement due to suboptimal resolution and sharpness.**

***Response***: Thank you for your careful review of our manuscript. In accordance with the recommendations, the TEM images were replaced with higher resolution images.

**8. For the WB analysis, it is imperative to incorporate a positive control to validate the protein bands' association with the isolated EVs.**

***Response***: Thank you for your careful review of our manuscript. The MISEV guideline recommends that when western blotting is used to characterize EVs, known antigen-positive and -negative controls should be included with the experimental samples whenever possible (J Extracell Vesicles. 2024 Feb;13(2):e12404). In this study, EVs were isolated from the bone marrow blood of 32 AML patients using a commercially available size exclusion chromatography method that has been widely cited and validated in previous studies. There was no obvious antigen-positive and -negative control that could be applied to all 32 patient samples from different time periods, and we had a limited amount of patient samples to perform experiments to determine this first. We also received a response from a size-exclusion chromatography company that β-actin, a common protein, is difficult to use as a control because it is fractionated when using the size-exclusion chromatography method. In addition, because the method used to isolate EVs was a commercially available size exclusion chromatography method that has been widely cited and validated in previous studies, only markers confirming that EVs were isolated were confirmed using western blotting. The rationale for selecting the commercial size-exclusion chromatography method used in this study, along with information on the referenced articles that used this approach, have been added to the manuscript (Page 9, lines 5–11).

**9. Include the complete membrane image as a supplementary figure in the WB analysis, not just a fraction of the identified protein.**

***Response***: Thank you for your careful review of our manuscript. Unadjusted blot images were uploaded in Supporting Information files.

**10. In the WB analysis, include the MW bands to confirm that the identified protein aligns with the expected molecular weight.**

***Response***: Thank you for your careful review of our manuscript. We modified the figure accordingly (**Figure 1D**).

**11. The authors are encouraged to incorporate a functional experiment involving a cell line, including an inhibitor for miR-188, miR-501, or a miR-181b mimic.**

**12. Additionally, a functional study or gene expression analysis should be included in the analyzed samples to validate the identified target genes.**

***Response***: Thank you for your careful review of our manuscript. In this study, we aimed to determine the potential of EV-derived miRNAs from the bone marrow blood of patients with AML as novel biomarkers. Mechanistic analysis of the candidate miRNAs and mRNAs identified in this study and their potential as druggable targets will be analyzed in follow-up studies.

**13. Regarding the statement in the discussion, 'Each miRNA can act on several mRNAs. In contrast, various mRNAs can interfere with a single miRNA,' it is recommended that the authors provide further clarification or expand on this concept, as it may be perceived as confusing in its current form.**

***Response***: Thank you for your careful review of our manuscript. The following sentences were written and added to the statement: "The central role of miRNAs is to regulate gene expression through canonical and non-canonical mechanisms. However, more than half of these processes are performed through non-canonical mechanisms, indicating that they are not always complementary". However, these sentences were deleted as they were considered to be confusing.

**Reviewer #3:**

This manuscript's considerable interest lies in identifying essential miRNAs by obtaining EVs from bone marrow samples of AML patients, and bioinformatics analysis methods have brought several meaningful potential regulatory relationships. However, it is necessary to mention that there is still more excellent value in this study that deserves the authors' reflection and additions, and some critical problems still need to be resolved.

**1. This study collected biological samples from 32 AML patients from 2008-2017, but information on sample collection, processing, and preservation is missing, and of particular concern is the difficulty of exosome identification if the samples have been preserved for an extended period, or in samples that have been preserved for 10 years. In addition, no clear instructions are given on the volume of samples to be collected per sample.**

***Response***: Thank you for your careful review of our manuscript. We incorporated the recommendations you provided into the manuscript (Page 7, lines 3–12).

**2. During the isolation of EVs, the authors used gel chromatography, which was a critical method in this study. The advantage of using SEC isolation is that the polymerization products of proteins and EVs are minimal. The functionality of the EVs is usually unaffected. However, it isn't easy to differentiate between particles of similar size but different characteristics by filtration or SEC separation methods based on component size. Thus, the authors should describe the reasons for choosing the SEC method concerning the purpose and needs of the study.**

***Response***: Thank you for your careful review of our manuscript. In the case of blood, especially plasma, interferences may be caused by lipophilic or hydrophilic proteins in the blood. To address this issue, we used commercial size-exclusion chromatography (EXo-I, Exopert, South Korea) with two-layer column beads consisting of CL-6B sepharose and sephacryl s-200 High Resolution to isolate EVs, which allows the exclusion of lipoproteins (ApoB) and soluble proteins (molecular weight, 35-75 kDa) from plasma while maintaining the integrity of EV markers. This method, which has been widely used in various studies, effectively separates EVs. Relevant content and literature were added to the manuscript (Page 9, lines 5–11).

**3. The target mRNAs of miRNAs have been established in several databases by experimental validation or computer prediction, and the data on the target genes of miRNAs available in different databases are not uniform, mainly in prediction methods, quantity, type, and elasticity of recorded data. Regarding the prediction of miRNA target genes, the authors used the TargetScan database, which is known to contain data based on the sequencing technique of 3P-seq to identify the corresponding 3'UTR region of a transcript and then to predict the target genes through the thermodynamic modeling of the interactions between the RNAs in conjunction with the analysis of the sequence comparisons. However, the authors can still try to apply more ways to explore the target genes of key miRNAs, for example, the multiMiRpackage, to obtain more prediction results and then carry out work such as miRNA bio-function or drug target analysis based on interaction analysis results.**

***Response***: Thank you very much for your careful review of our manuscript. Although there are many analysis methods related to mRNA-miRNA interactions, we used the TargetScan database, which is used as a de facto standard. It would be good to use many databases, but due to the heterogeneous patterns of each database, we preferred to use one database in this study. We are using different mRNA-miRNA databases to propose diagnostic markers and treatment strategies for AML and plan to further investigate the heterogeneity between different databases in the future.

**4. The authors performed survival analyses based on clinical data from TCGA-LAML, suggesting that the list reports basic clinical information for the 174 AML patients used in this study.**

***Response***: Thank you for your careful review of our manuscript. Relevant content was presented as S4_TCGA_AML_174_patients in Supporting Information files. Relevant information was also included in the manuscript (Page 21, line 4).

**5. The authors extracted exosomes and performed exosome particle size and marker assays in the study. Still, since SEC is a separation methodology approach based on size and the authors used two layers of column beads consisting of CL-6B sepharose and sephacryl s-200 high resolution to try to avoid the possibility of other contaminants of similar sizes that were present in the separation process, then the authors should have reported the results of the exosome concentration data.**

***Response***: Thank you for your careful review of our manuscript. Relevant content was added to the manuscript, and to **Figure 1A** and the corresponding legend.

**6. By differential expression analysis of EVs-derived miRNAs, the authors obtained 965 EVs-derived miRNAs, but the authors listed only 32 DEmiRNAs in the non-favorable risk group, which is incomplete, and it is recommended that the authors provide the data results of all the differentially expressed miRNAs or the expression matrix of the sequencing results of the miRNAs in the form of supplemental material. Similarly, the up-and down-regulated significant miRNAs could be presented as figures based on the**

**ggplot2 or heatmap package.**

***Response***: Thank you for your careful review of our manuscript. An expression matrix containing the total fold change and p-value for 965 items was presented in S2_AML_RPM_Genotype and S3_AML_RPM_Heatmap in Supporting Information files. Relevant information was also included in the manuscript (Page 15, line 10–11).

**7. Presenting the results of survival analyses in table form is encouraged, but attention needs to be paid to the form and readability of the table, and a formal revision of Table 3 and Table 4 is needed.**

***Response***: Thank you for your valuable comment. Although we discussed changing the format of the table and splitting it into several supplemental tables, it was also not optimal to present the results with these format changes. Therefore, we have retained the current format of the table but modified its content to improve the readability of the information regarding the highlighted miRNAs and mRNAs in this study (**Tables 3 and 4**).

**8. The authors could have carried out more analyses on the screened key miRNAs, and the research methods provided in the current study may be too simplistic in the bioinformatics section. It is recommended to add, for example, analyses of GO, KEGG, Reactome, drug prediction, immune infiltration, and other multiple biological regulatory functions for miRNAs and the target genes or to increase the level of the analysis by further constructing the clinical prediction model based on the miRNAs to emphasize the biological significance of the key miRNAs.**

***Response***: Thank you for your careful review of our manuscript. We have already performed several biological regulatory functional analyses for miRNAs and the target genes, such as GO, KEGG, etc., but there was no meaningful information to present. We did not present it as supplementary material because it might obscure the point of this study.

**9. Regarding the discussion section of the study, the authors are required to summarise and analyze the important results found to highlight the significance of this study. As it is well known that the exosomes of AML cells have aberrant biogenesis, secretion, and uptake, also the functions are of great interest, especially in the context of the exosomes of AML stem cells in creating an adaptive bone marrow microenvironment, tumor resistance, and extramedullary infiltration, the authors still need to address the contributions of the study and future challenges in the context of their important findings.**

***Response***: Thank you for your careful review of our manuscript. We added a paragraph to the Discussion section to address the function of EVs in the crosstalk between AML and the microenvironment, AML stem cell maintenance, proliferation, progression, and chemotherapy resistance (Page 28, lines 17–23; and page 29, lines 1–14). We also briefly summarized these points before concluding to emphasize the importance of EVs in AML (Page 31, lines 13–17).
