# Supplementary material for: Use of extracellular vesicle microRNA profiles in patients with acute myeloid leukemia for the identification of novel biomarkers
Source: PLoS One. 2024 Aug 23;19(8):e0306962. doi: 10.1371/journal.pone.0306962 (PMC11343415; doi:10.1371/journal.pone.0306962)
Supplement: Attachment — Submitted filename: 0_Response_to_reviewers_20240604.docx [file pone.0306962.s011.docx]

**Manuscript ID: PONE-D-23-13124R1**

**Title:** Use of extracellular vesicle microRNA profiles in patients with acute myeloid leukemia for the identification of novel biomarkers

Dear Prof. George Vousden:

We appreciate your assistance in processing our manuscript and the insightful comments and suggestions provided by the editor and reviewers. We have incorporated the suggested modifications and included them below. Additionally, our manuscript has been reviewed by an English-speaking editor. Thank you for your guidance, and we hope to hear from you soon regarding the acceptance of our manuscript for publication.

Sincerely yours,

Yong Park, MD, Ph.D.

Department of Internal Medicine, Korea University College of Medicine 73 Goryeodae-ro, Seongbuk-gu, Seoul 02841, Republic of Korea

Tel: +82-2-920-6847, Fax: +82-2-920-6520, E-mail: [paark76@hanmail.net](mailto:paark76@hanmail.net)

**List of reviewers’ comments and point-by-point responses**

**In each response, page and line numbers refer to the revised manuscript.**

**In the revised manuscript, text changes are highlighted in yellow.**

**Editor:**

**1. Please provide answer to requested analysis and its impact as per study main objective.**
***Response***: According to the reviewer, we have included the analysis and described the relevant content in the manuscript (Page 15, lines 15–19).

**2. Please also ensure that the submitted information is readible as tables or figures (S2_AML_RPM_Genotype).**

***Response***: Sorry for the confusion. We have converted the format of the file to an Excel file so that you can review the data in each column (S2_AML_RPM_Genotype).

**Reviewer #1:**

**1. The Authors partially reply to my concerns. In particular, no analysis in the favorable group was made. The reply of the Authors is not sufficient. The favorable group could be considered in order to define a "protective" setting of miRNA. Moreover, despite the consideration that the samples were collected at diagnosis, the impact of the information must not be limited at that time. miRNA are considered as predictive of different variables, included the response to therapy. A correlation between the basal level of miRNA expression and the response to therapy/HSCT is required.**

***Response***: We would like to express our gratitude for your valuable feedback. Additional analysis of differentially expressed EV-derived miRNA was conducted for patients who had achieved complete remission following induction chemotherapy or who had survived for more than one year following stem cell transplantation (**Supplementary figure 1 and 2,** **S4_AML_RPM_CR_after_induction_therapy, and S5_AML_RPM_Transplantation in Supporting Information files**). Unfortunately, the number of bone marrow samples from patients who underwent transplantation was too small to yield significant results. Although the miRNA expression landscape based on the achievement of complete remission following induction chemotherapy showed relatively distinct patterns, we were unable to validate these findings due to the lack of corresponding information in the TCGA dataset. We have included the additional analysis and related files in the manuscript and presented them as supplementary files (Page 15, lines 15–19).

**Reviewer #3:**

**1. The authors seem to have done their best to address my comments, and in subsequent studies I hope that the authors will carry out the appropriate biofunctional analyses for the key miRNAs they have found.**

***Response***: We are grateful for your valuable input. Moving forward, we will be committed to conducting the necessary biofunctional analyses for the key miRNAs identified in subsequent studies.
